# Aberrant Pregnancy Adaptations in the Peripheral Immune Response in Type 1 Diabetes: A Rat Model

**DOI:** 10.1371/journal.pone.0065490

**Published:** 2013-06-21

**Authors:** Bart Groen, Thera P. Links, Joop D. Lefrandt, Paul P. van den Berg, Paul de Vos, Marijke M. Faas

**Affiliations:** 1 Department of Endocrinology, University Medical Center Groningen and University of Groningen, The Netherlands; 2 Department of Internal Medicine, Division of Vascular Medicine, University Medical Center Groningen and University of Groningen, The Netherlands; 3 Department of Obstetrics and Gynecology, University Medical Center Groningen and University of Groningen, The Netherlands; 4 Department of Pathology and Medical Biology, Division of Medical Biology, University Medical Center Groningen and University of Groningen, The Netherlands; Medical Faculty, Otto-von-Guericke University Magdeburg, Medical Faculty, Germany

## Abstract

**Introduction:**

Despite tight glycemic control, pregnancy complication rate in type 1 diabetes patients is higher than in normal pregnancy. Other etiological factors may be responsible for the development of adverse pregnancy outcome. Acceptance of the semi-allogeneic fetus is accompanied by adaptations in the maternal immune-response. Maladaptations of the immune-response has been shown to contribute to pregnancy complications. We hypothesized that type 1 diabetes, as an autoimmune disease, may be associated with maladaptations of the immune-response to pregnancy, possibly resulting in pregnancy complications.

**Methods:**

We studied pregnancy outcome and pregnancy-induced immunological adaptations in a normoglycemic rat-model of type 1 diabetes, i.e. biobreeding diabetes-prone rats (BBDP; 5 non-pregnant rats, 7 pregnant day 10 rats and 6 pregnant day 18 rats) , versus non-diabetic control rats (i.e. congenic non-diabetic biobreeding diabetes-resistant (BBDR; 6 non-pregnant rats, 6 pregnant day 10 rats and 6 pregnant day 18 rats) and Wistar-rats (6 non-pregnant, 6 pregnant day 10 rats and 5 pregnant day 18 rats)).

**Results:**

We observed reduced litter size, lower fetal weight of viable fetuses and increased numbers of resorptions versus control rats. These complications are accompanied by various differences in the immune-response between BBDP and control rats in both pregnant and non-pregnant animals. The immune-response in non-pregnant BBDP-rats was characterized by decreased percentages of lymphocytes, increased percentages of effector T-cells, regulatory T-cells and natural killer cells, an increased Th1/Th2-ratio and activated monocytes versus Wistar and BBDR-rats. Furthermore, pregnancy-induced adaptations in BBDP-rats coincided with an increased Th1/Th2-ratio, a decreased mean fluorescence intensity CD161a/NKR-P1b ratio and no further activation of monocytes versus non-diabetic control rats.

**Conclusion:**

This study suggests that even in the face of strict normoglycemia, pregnancy complications still occur in type 1 diabetic pregnancies. This adverse pregnancy outcome may be related to the aberrant immunological adaptations to pregnancy in diabetic rats.

## Introduction

Type 1 diabetes mellitus (T1D) during pregnancy is associated with adverse pregnancy outcome, such as pre-eclampsia, prematurity, macrosomia and perinatal death [1], [2]. Up to now, this has been attributed to frequent episodes of hyperglycemia [3], [4]. This argumentation was supported by the observation that improved metabolic control before and during pregnancy in women with type 1 diabetes decreased the number of pregnancy complications. However, even under adequate glycemic control, complication rate is still increased [1], [5], suggesting that other etiological mechanisms may be involved. A possible candidate may be immunological changes associated with this autoimmune disease in these women [6], [7].

Acceptance of the semi-allogeneic fetus during normal pregnancy is facilitated by adaptations in the maternal peripheral and local immune-responses [7]. Peripherally, innate immune cells become activated during pregnancy as characterized by upregulation of various activation markers on monocytes and granulocytes and changes in cytokine secretion [8], [9]. The specific immune-response shifts from a type 1 (i.e. cellular; Th1) immune-response towards a type 2 (i.e. humoral; Th2) immune-response [10], [11]. The adaptations at the local level are accompanied by an influx of Natural Killer (NK) cells into the decidua. Further, macrophages infiltrate the decidua as well and differentiate towards the M2 phenotype, which have immunosuppressive properties [12], [13]. Altered adaptations in these immune-responses to pregnancy may induce maternal and perinatal complications, like pre-eclampsia, pregnancy-induced hypertension, intrauterine growth retardation, preterm delivery and/or abortion [14]–[18]. Disturbances of the immune response at the local level may lead to impaired placentation, resulting in, for instance, pre-eclampsia [19]. Disorders of the immune response at the peripheral level are linked to the development of (cardio)vascular diseases, such as hypertension [20].

Type 1 diabetes is an auto-immune disease with many immunological changes as compared to healthy individuals; previous studies showed a shift towards a Th1 immune-response, decreased number and/or function of Treg [21], decreased number of NK cells [22], [23], and activation of monocytes [24] in patients with type 1 diabetes versus healthy individuals. The question therefore rises if the immune-response of type 1 diabetic patients is able to face the necessary immunological changes during pregnancy. Hence, we hypothesized that in type 1 diabetes the immunological adaptations to pregnancy might be disturbed and therefore contribute to higher frequency of maternal and perinatal pregnancy complications.

To test our hypothesis we applied a rat-model of type 1 diabetes, the biobreeding diabetes-prone (BBDP) rat, to study adaptations of the peripheral immune-response to pregnancy. This model is generally accepted as a model for type 1 diabetes and well controlled in terms of immunological changes and lack overshadowing secondary effects of medication and heterogeneity in responses in human population. Moreover, BBDP-rats were kept strictly normoglycemic during pregnancy using insulin pellets, to avoid the influence of hyperglycemia. We used congenic non-diabetic biobreeding diabetes-resistant (BBDR) and Wistar-rats (as the parent strain of BBDP and BBDR-rats) as controls.

## Materials and Methods

### Experimental design and animals

**Figure 1:**
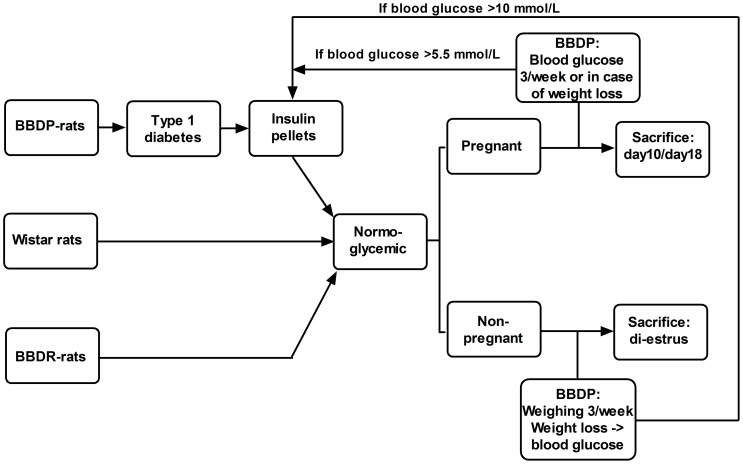
Flowchart of the experimental design.

Approval of the institutional Animal Care Ethics Committee, University of Groningen (application-number DEC-5759a) was obtained; all animals received human care in compliance with Dutch Law on Experimental Animal Care. Figure 1 shows the experimental design. Female rats of three different strains were used: Biobreeding diabetes-prone (BBDP) rats, which develop type 1 diabetes spontaneously between 60–120 days after birth; animals were only used after diabetes was established. Congenic biobreeding diabetes-resistant (BBDR) rats which do not spontaneously develop diabetes and act, therefore, as a control model. However, since BBDR-rats are sensitive to diabetes induction due to viral infection, depletion of regulatory T cells or Toll-like receptor ligation [25], we used Wistar (W) rats, the parent strain of BBDP and BBDR-rats [25], which are not sensitive to diabetes induction, as our absolute controls. BBDP and BBDR-rats were bred and nurtured at the Central Animal Facility of the UMCG [26]. All rats (age 3–6 months and weighing 200–250 gr) were kept in temperature- and light-controlled environment (lights on from 6 AM to 6 PM). Vaginal smears were taken daily to assess ovarian cyclicity. Pregnancy was achieved by housing female rats on the night of pro-estrus with a fertile male. The next day, when spermatozoa were detected in the smear, was pregnancy day 0. Non-pregnant animals were sacrificed on di-estrus.

BBDP-rats were weighed three times a week from 50 days of age. After a decrease in bodyweight, glucose was measured in tail-vein blood using a glucose-sensor (Accu-Check Sensor Comfort, Roche Diagnostics Nederland B.V., Almere, The Netherlands)). After establishing diabetes in BBDP-rats (blood glucose >15 mmol/L), rats received a half insulin implant (Linplant-sustained-release-insulin-implant; LinShin Canada Inc, Toronto, Canada) subcutaneously, to maintain blood glucose <5.5 mmol/L. To prevent hypoglycemia due to an excess amount of insulin, we implanted a half insulin implant, which released about 1 unit/24 hr. In our experience half an insulin implant will keep the BBDP-rats normoglycemic for 3–4 weeks. These insulin treated non-pregnant diabetic rats were weighed 3 times a week and blood glucose was measured at least once every 3 weeks as well as in case of weight loss. Blood glucose was always between 4.5 and 5.5 mmol/L, except when weight loss occurred, which was always associated with hyperglycemia. An additional half insulin pellet was implanted if glucose rose again >10 mmol/L. Due to maternal weight gain during pregnancy, we could not only rely on maternal weight gain for estimating blood glucose. Therefore, apart from measuring maternal weight gain every day, blood glucose of pregnant diabetic rats was measured 3 times a week. Also in these animals, blood glucose remained between 4.5 and 5.5 mmol/L, lack of weight gain as well as weight loss was associated with hyperglycemia. If blood glucose rose above 5.5 mmol/L, rats received an additional half insulin implant.

Power analysis using the general variance of immune parameters in Wistar rats (i.e. about 10%) and a physiologically relevant effect size of 20% with α = 0.05 and ß = 0.8, revealed an n = 5 per group. We included 6 rats in each group. Unfortunately, 1 pregnant W rat appeared to be pseudopregnant rather than pregnant and was excluded from the study, and 1 non-pregnant BBDP rat turned out to be pregnant and was moved to the pregnant group). For all rats strains we included non-pregnant rats (BBDP: n = 5; W: n = 6; BBDR: n = 6), and pregnant rats at day 10 (BBDP: n = 7; W: n = 6; BBDR: n = 6) and at day 18 (BBDP: n = 6; W: n = 5; BBDR: n = 6). At the day of sacrifice, rats were anesthetized with isofluorane/O_2_, and blood was collected from the aorta into 10 ml EDTA tubes (BD-Plymouth, UK). We counted number of implantations sites for day 10 (d10) pregnant rats. For day 18 (d18) pregnant rats, we counted number of viable fetuses and number of resorptions, weighed individual placentas and fetuses and checked these for major abnormalities.

### Sample handling

#### White Blood Cell (WBC) counts

Twenty µl of blood was diluted in 500 µl PocH-buffer and leukocytes were counted using a microcell counter (Sysmex PocH 100i, Sysmex Netherlands).

#### Reagents

The following reagents were used: Washing-buffer (phosphate-buffered saline (PBS) with 0.5% bovine serum albumin and 0.1% NaN_3_), FACS^TM^ lysing buffer solution (BD Biosciences, Breda, the Netherlands), FoxP3-staining buffer set (eBioscience, Vienna, Austria), complete RPMI-1640 medium (Lonza Benelux, Breda, the Netherlands) supplemented with 60 µg/ml gentamycin (Invitrogen, Breda, the Netherlands), Lymphoprep (Axis-shield PoC-As, Oslo, Norway), TRIzol Reagent (Invitrogen), Absolute QPCR ROX Mix (Westburg, Leusden, the Netherlands) and RNase-free water (Qiagen, Hilden, Germany).

#### Antibodies

The following antibody-cocktails were used to stain different leukocytes subsets and their activational status. Unless stated otherwise, they were purchased from BioLegend (BioLegend Europe, Uithoorn, the Netherlands).

In the T-lymphocyte cocktail the following antibodies were used: Mouse-anti-rat CD3 Biotin-labeled (clone eBioG4.18; eBioscience, Frankfurt, Germany), mouse-anti-rat CD4 AlexaFluor700-labeled (clone W3/25; AbD Serotec, Germany), mouse-anti-rat CD25 FITC-labeled (clone Il-2R; BD Pharmingen, Breda, Netherlands), Rat-anti-mouse/rat FoxP3 APC-labeled (clone FJK, eBioscience) and Rat-APC-labeled isotype control IgGG2a (clone eBR2a, eBioscience) and PerCP-labeled streptavidin.

In the Natural Killer cells cocktail the following antibodies were used: Mouse-anti-rat NKRP1a/CD161a PE-labeled (clone 10/78; BD Pharmingen), Mouse-anti-rat NKRP1b Biotin-labeled (clone STOK27: Kindly donated by J.T. Vaage, Institute of Immunology, Rikshospitalet University Hospital, Oslo, Norway) and PerCP-labeled streptavidin.

In the monocytes cocktail the following antibodies were used: Mouse-anti-rat CD172a PE-labeled (clone OX-41), Mouse-anti-rat CD43 AlexaFluor647-labeled (clone W3/13) and FITC-labeled anti-rat CD4 (clone OX-35; BD Pharmingen).

#### Sample labeling

Immediately after sampling, 1200 μl whole blood was mixed with 1200 μl RPMI-1640 after which samples were aliquoted (200 μl per tube) into eleven tubes (six for compensation of the flowcytometry, 2 for lymphocytes/lymphocyte isotype cocktail, 1 for NK-cell cocktail, 2 for monocytes/monocytes isotype cocktail) followed by centrifugation and aspiration of plasma. Subsequently, all tubes were incubated with antibodies (-cocktails) in dark for 30 min and washed. Thereafter, tubes were either incubated with PerCP-labeled streptavidin or with washing-buffer for 15 minutes in dark followed by lysis of red blood cells (RBC) using lysing buffer for 30 minutes in the dark. After centrifugation, aspiration and washing, tubes for lymphocyte/lymphocyte isotype were incubated with 200 μl fixperm, while 300 μl washing-buffer was added to the other tubes which were stored at 4°C in dark. After 30 min incubation at RT in dark, tubes for lymphocyte/lymphocyte isotype were washed with 200 μl perm followed by incubation with FoxP3 antibody or its isotype for 30 min at RT in dark and washed with 1 ml perm afterwards. Finally, 300 μl washing-buffer was added to these tubes and tubes were stored at 4°C in dark. Flow cytometry was done within 24 hr.

### Flow cytometry

Two hundred fifty thousand events were counted using the BD^TM^ LSR II flow cytometer (BD Biosciences), and data were saved for later analysis. Analysis was performed using FlowJo 7.6.1 (Tree Star, Inc., Ashland, OR, USA).

#### Differential cell counts

First, we assessed percentages of different leukocyte populations. A gate was set around leukocytes in the forward/sideward (FSC/SSC) scatter-plot, which was copied to a CD43/CD172a plot. Lymphocytes are CD172a negative. Although monocytes and granulocytes are both CD172a positive, monocytes show a stronger CD172a expression and weaker CD43 expression as compared to granulocytes. Therefore, this plot allowed us to select monocytes (CD172a^++^/CD43^−/+^), granulocytes (CD172a^+^/CD43^+^) and lymphocytes (CD172a^−^). Percentages of these subpopulations from total leukocyte population were calculated.

#### Lymphocytes

**Figure 2:**
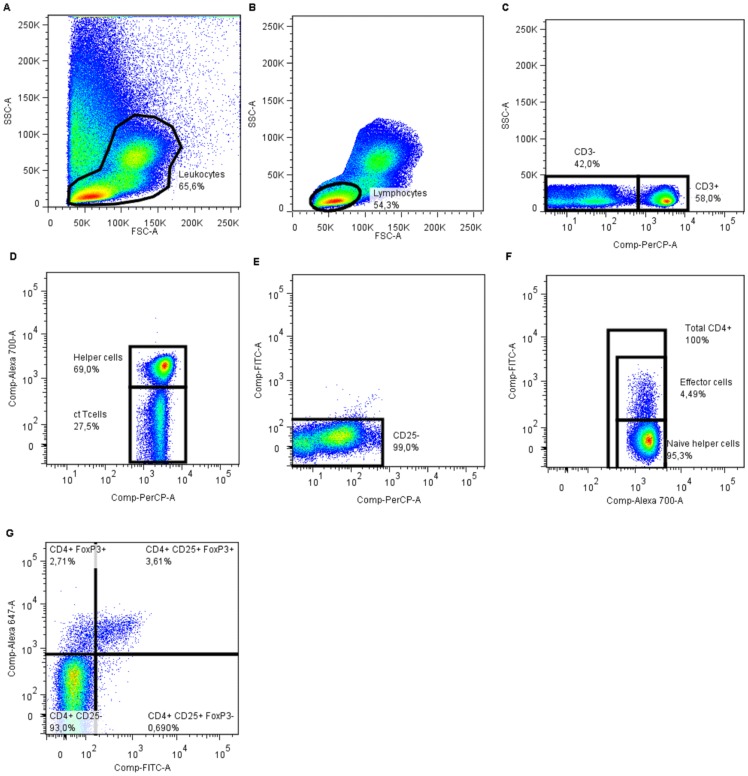
An representative example of a FACS analysis procedure to evaluate lymphocytes subpopulations. [A] A forward/sideward (FSC/SSC) scatterplot of all events in which leukocytes were identified. [B] The leukocyte gate was copied to a FSC/SSC scatterplot in which the lymphocytes are identified. [C] These lymphocytes are copied to a SSC/CD3-PerCP scatterplot to distinguish T-lymphocytes (CD3^+^). [D] The lymphocytes are further divided into helper (CD3^+^/CD4^+^) and cytotoxic (CD3^+/^CD4^+^; ct) T-cells in a CD3 PerCP/CD4 Alexa 700 scatterplot. [E] A CD3 PerCP/CD25 FITC scatterplot in which CD3^−^ cells were copied to assess the gate for FITC-positivity. [F] This gate for FITC positivity was copied to a CD4 Alexa 700/CD25 FITC scatterplot to identify CD25 positive and negative cells; i.e. effector (CD3^+^/CD4^+^/CD25^+^) and naive helper T-cells (CD3^+^/CD4^+^/CD25^−^). [G] A CD25 FITC/FoxP3 Alexa 647 scatterplot to identify the regulatory T-cell (CD3^+^/CD4^+^/CD25^+^/FoxP3^+^; Treg) positive population.

To identify percentages of various lymphocyte populations, a gate was around leukocytes in a FSC/SSC plot (Figure 2A). This gate was copied to a new FSC/SSC plot and lymphocytes were identified (Fig. 2B). These lymphocytes were copied into a SSC/CD3 graph to identify T-lymphocytes (SSC/CD3^+^; Fig. 2C). This T-lymphocyte gate was copied to a CD3/CD4 scatter-plot (Fig. 2D). Subsequent gates were set on CD3^+^/CD4^+^ lymphocytes (T-helper lymphocytes; Th) and the CD3^+^/CD4^−^ population (T-cytotoxic lymphocytes; Tc). The Th/Tc ratio was calculated by dividing the percentage Th by the percentage Tc. To distinguish CD25 positive and negative cells, gates were set based on FITC-positivity of CD3^−^ cells in such a way that 99% of these cells were negative for CD25 (Fig. 2E); the CD3^+^/CD4^+^ gate was copied to a CD4/CD25 scatter-plot (Fig. 2F). The populations CD3^+^/CD4^+^/CD25^+^ and CD3^+^/CD4^+^/CD25^−^ were identified as effector T-lymphocytes (Teff) and naive helper cells respectively. Regulatory T-cells (Treg) were identified as CD3^+^/CD4^+^/CD25^+^/FoxP3^+^ (Fig. 2G).

#### NK-cells

To identify NK-cells, a gate was set around leukocytes in a FSC/SSC plot. This gate was copied to a new FSC/SSC plot to identify lymphocytes. The lymphocyte gate was copied to a CD161a/SSC plot (CD161a or NKR-P1a is an activating receptor and present on all NK-cells); a gate was set on CD161a^+^. These cells were identified as NK-cells. Further, mean fluorescence intensity (MFI) of this stimulatory receptor CD161a and MFI of the inhibitory receptor (i.e. NKR-P1b) on these NK-cells was assessed. The MFI ratio of CD161a/NKR-P1b reveals the balance of stimulatory/inhibitory receptors.

#### Monocytes

To analyze monocyte subsets, a gate was set around leukocytes in a FSC/SSC plot. This gate was copied to a CD43/CD172a plot, and a subsequent gate was set on CD172a^++^ cells (monocytes). This gate was copied to a new CD172a/CD43 plot and gates were set on CD172a^++^/CD43^+^ and CD172a^++^/CD43^++^ (classical and non-classical monocytes, respectively). The percentages of CD4 positive cells and CD4 MFI were measured in each subpopulation to analyze activation-status of these subpopulations.

### Messenger-RNA (mRNA) analysis

The remainder of the blood sample was diluted 1:1 with PBS and peripheral blood mononuclear cells (PBMC) were isolated using lymphoprep. After washing, the PBMC’s were resuspended in 1ml of TRIzol and frozen at −80°C until further analysis. Total RNA was extracted by chloroform-isopropanol according to the manufacturer’s protocol. cDNA was reverse transcribed using Superscript-II reverse-transcriptase kit (Invitrogen Life Technologies) according to manufacturer’s protocol.

Taqman Gene Expression Assays (Applied Biosystems, Carlsbad, CA, USA) were used. Three housekeeping genes, i.e. 1) β-Actin (Rn00667869_m1), 2) B2M (Rn00560865_m1) and 3) GAPDH (Rn01775763_g1) and three genes were assessed, i.e. 1) Tbx21 (Th1 transcription-factor; Rn01461633_m1), 2) GATA-3 (Th2 transcription-factor; Rn00484683_m1) and 3) ROR-C (Th17 transcription-factor; Rn01533717_g1). Real-time RT-PCRs were performed in triplicate in 20 μl according to Applied Biosystems protocol. Runs were performed by a 7900HT Fast real-time PCR system (Applied Biosystems), under standard conditions. Variance of housekeeping genes was tested using NormFinder [27]. As GAPDH showed the least variance between non-pregnant and pregnant groups, mRNA data were normalized to GAPDH mRNA using ΔCt = Ct_GENE OF INTEREST_ – Ct_GAPDH_. Gene data are expressed as 2^−ΔCt^. The Th1/Th2 ratio was calculated by dividing Tbx21 gene expression by GATA-3 gene expression.

### Statistics

Continuous parameters were expressed as median (Q_1_–Q_3_). To compare differences between the 3 non-pregnant strains of rats, Mann-Whitney U-test was used. To evaluate pregnancy-induced differences, linear regression analysis was performed. The slope of the regression line through data of non-pregnant, pregnant day 10 and 18 was calculated and it was tested (by analyzing the covariance; ANCOVA) whether the slope was significantly different from zero or significantly different between different strains. P-values of <0.05 were considered as statistically significant and p-values between 0.05–0.1 as a trend. Bonferroni correction was used to correct for multiple comparisons. In order to show pregnancy effects, median of non-pregnant parameters was set at 100% and percentages from day 10 and day 18 were recalculated from non-pregnant data using ratios and proportions (a/b  =  c/d). Statistical analyses were performed using PASW for Windows version 18 (SPSS, Inc., Chicago, IL, USA) and GraphPad Prism 5 for windows (GraphPad Software, Inc., La Jolla, Ca, USA).

## Results

### Higher frequency of fetal complications in BBDP-rats

We first evaluated fetal weight and resorptions in normoglycemic BBDP-rats and our controls. A trend towards decreased number of implantation sites was found in pregnant d10 BBDP-rats as compared to d10 W-rats (Table 1). At d18, number and weight of viable fetuses and placentas was significantly lower and number of resorptions was significantly higher in BBDP-rats as compared to W-rats. With respect to the pregnancy outcome of BBDR-rats, the results of this strain were between BBDP and W-rats.

**Table 1:** Basic characteristics of the different rat strains.

	BBDP		Wistar		BBDR	
	d10 (n = 7)	d18 (n = 6)	d10 (n = 6)	d18 (n = 5)	d10 (n = 6)	d18 (n = 6)
Number of implantation sites/viable fetuses	8.0(3.0–10.0)	6.0(4.0–8.0)	14.0(8.3–14.3)†	12.0(11.0–15.0)*	9.5(4.8–10.8)	8.5(6.3–12.0)§
Number of resorptions	n/a	2.5(0.8–3.0)	n/a	0.0(0.0–0.0)*	n/a	0.0(0.0–1.0)†
Maternal weight gain (gr.)	19.0(14.0–26.0)	59.0(56.5–64.3)	39.5(34.5–43.3)*	100.0(87.5–109.0)*	23.5(20.3–26.0)‡	70.5(60.8–77.0)‡
Weight of pups (gr.)	n/a	0.896(0.867–0.993)	n/a	1.177(1.124–1.239)*	n/a	1.087(1.065–1.108)* ‡
Placental weight (gr.)	n/a	0.371(0.313)	n/a	0.454(0.424–0.550)*	n/a	0.475(0.384–0.512)†

Values are expressed as median (Q_1_–Q_3_). Abbreviations: d10 = sacrificed at day 10 of pregnancy, d18 = sacrificed at day 18 of pregnancy.

* : significantly different from the BBDP-rats at the same gestational age (Mann-Whitney U-test; p<0.05).

† : statistical trend as compared with BBDP-rats at the same gestational age (Mann-Whitney U-test; p = 0.10–0.05).

‡ : significantly different from W-rats at the same gestational age (Mann-Whitney U-test; p<0.05).

§ : statistical trend as compared with W-rats at the same gestational age (Mann-Whitney U-test; p = 0.10–0.05). The immune-response of non-pregnant BBDP-rats.

To assess pregnancy-induced adaptations of the immune-response, we first evaluated the immune-response in the 3 non-pregnant rat strains; this is the immune-response at fertilization. In general, our main focus was on differences between BBDP and W-rats, since W-rats are our absolute controls. However our graphs also show comparisons between BBDR-rats and BBDP or W-rats.

### Decreased WBC and lymphocytes in BBDP-rats

WBC counts and percentages of different leukocyte subsets were significantly lower in non-pregnant BBDP-rats as compared to W-rats (Table 2).

**Table 2:** Distribution of the different leukocyte subsets in non-pregnant and pregnant animals.

	BBDP			Wistar			BBDR		
	NP	d10	d18	NP	d10	d18	NP	d10	d18
WBC (x10^9^ cells/L)	***2.1 (1.8–2.4)***	***1.8 (1.2–2.2)***	***3.3 (3.0–4.9)***	3.9 (3.0–5.3)	4.5 (3.7–6.8)	4.4 (4.0–5.8)	2.9 (2.7–5.1)	3.2 (2.6–3.6)	3.7 (3.5–3.9)
Lymphocytes	***59.5 (39.5–77.6)***	***41.5 (38.8–49.9)***	***25.7 (22.2–35.3)***	84.4* (80.1–86.2)	***83.8 (79.1–87.7)***	***65.9 (63.7–70.1)***	***80.1 (78.0–83.0)***	***76.5 (73.0–79.3)***	***68.6 (64.3–71.2)***
Monocytes	15.0 (10.6–26.9)	23.6 (16.4–25.8)	22.3 (20.6–24.7)	7.2 (6.6–8.1)	8.0 (6.6–9.7)	8.4 (6.7–10.2)	***9.8*** **^†^** *** (8.8–10.5)***	***12.5 (10.8–15.6)***	***16.9 (15.8–17.5)***
Granulocytes	***25.5 (11.8–33.6)***	***33.9 (26.4–44.8)***	***50.6 (43.0–55.5)***	***8.6 (6.7–12.7)***	***7.5 (6.5–11.1)***	***25.7 (22.3–27.0)***	10.1* (8.9–11.9)	***11.1 (9.7–12.7)***	***14.7 (12.5–18.9)***

Values are expressed as median (Q_1_–Q_3_). Due to technical problems, WBC’s of only 2 NP BBDP rats were available. Abbreviations: NP = non-pregnant, d10 = sacrificed at day 10 of pregnancy, d18 = sacrificed at day 18 of pregnancy. *: significantly different from non-pregnant BBDP rats (Mann-Whitney U-test, p<0.05). †: significantly different from non-pregnant W rats (Mann-Whitney U-test, p<0.05). Bold and italic displayed values indicate a slope significantly different from zero during the course of pregnancy (Linear regression test, ANCOVA; p<0.05).

### Increased percentage of effector and regulatory T-cells and NK-cells in non-pregnant BBDP-rat

**Figure 3:**
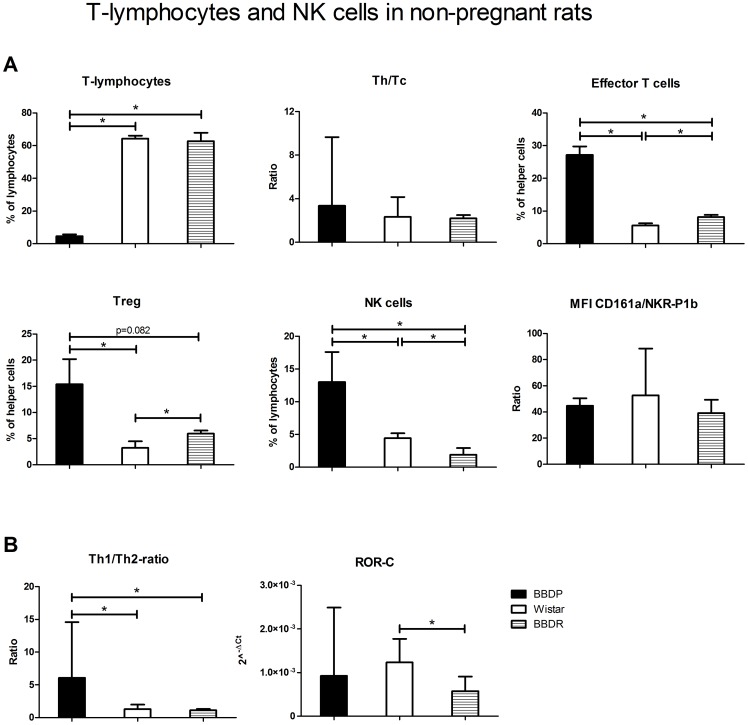
Lymphocyte subpopulations in non-pregnant rats. [A] The percentages of T-lymphocytes (of the total leukocyte population), the ratio of T-helper cells (Th) and cytotoxic T-cells (Tc), percentages effector T-cells (of Th; Teff), regulatory T-cells (of Th; Treg), NK-cells (of total lymphocyte population) and the ratio of MFI CD161a/NKR-P1b of NK-cells of the three non-pregnant rat-strains. [B] The Th1/Th2-mRNA ratio and mRNA expression of ROR-C of the three non-pregnant rat-strains. Values are expressed as median with range (Q_1_–Q_3_), mRNA was expressed as Fold Change (2^∧−ΔCT^). ‘*’ significant difference, Mann-Whitney U-Test, p<0.05.

Although the percentage of T-lymphocytes is decreased in BBDP-rats vs. W-rats, the Th/Tc ratio was not affected (Fig. 3A). Moreover, we observed an increased percentage of effector T-cells (and a decreased percentage of naïve T-cells (not shown)) and regulatory T-cells (Treg) in BBDP vs. W-rats. Also NK-cell numbers were different between the strains: the percentage of NK-cells was increased in BBDP-rats as compared to W-rats, although the MFI ratio CD161a/NKR-P1b was similar between these strains.

### Increased Th1/Th2 ratio in non-pregnant BBDP-rats

Since we found differences in T-cell frequencies between BBDP-rats and W-rats, we evaluated the Th1-, Th2- and Th17-response, which are important for healthy pregnancy, by measuring transcription factors specific for Th1 (Tbx21), Th2 (GATA-3) and Th17 (ROR-C) cells (Fig. 3B). The ratio Tbx21/GATA-3 mRNA expression was significantly higher in BBDP-rats vs. W-rats. This was due to increased mRNA expression of Tbx21 in BBDP-rats as compared to W-rats, while a trend towards decreased GATA-3 expression was found in BBDP-rats versus W-rats (results not shown). ROR-C mRNA did not differ between BBDP and W-rats, but was significantly lower in BBDR vs. W-rats.

### Activation of monocytes in non-pregnant BBDP-rat

**Figure 4:**
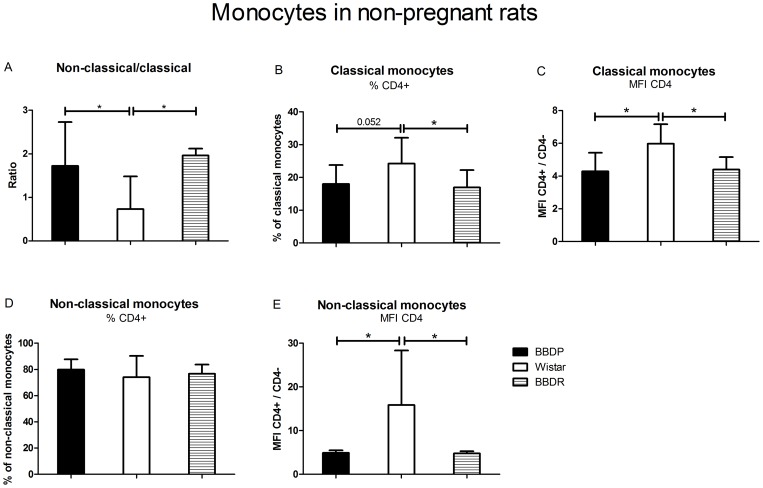
Monocyte subpopulations in non-pregnant rats. The ratio of non-classical/classical monocytes [A], the percentage of CD4^+^ classical monocytes [B], the MFI of CD4 on classical monocytes [C], the percentage of CD4^+^ non-classical monocytes [D] and the MFI of CD4 on non-classical monocytes [E] in non-pregnant animals of each strain. Values expressed as median with range (Q_1_–Q_3_), ‘*’ significant difference, Mann-Whitney U-Test, p<0.05.

Since monocytes play an important role in immunological adaptations during pregnancy, we also studied monocyte subsets and activation. We observed a higher non-classical/classical monocyte ratio in BBDP than in W-rats (Figure 4). This was due to a lower percentage of classical monocytes and a higher percentage of non-classical monocytes in BBDP vs. W-rats (not shown). The monocyte activation status was further evaluated by expression of CD4, which decreases upon activation [28]. Percentage of CD4^+^ classical monocytes tended to be lower in BBDP vs. W-rats. Moreover, MFI of CD4 on classical and non-classical monocytes was decreased in BBDP-rats as compared to W-rats.

### Pregnancy-induced immune changes in BBDP-rats

#### Pregnancy-induced changes in leukocyte subsets are more pronounced in BBDP-rats

In BBDP-rats, WBC-count significantly increased during the course of pregnancy (slope 0.103·10^9^; p = 0.03) (Table 2). This appeared to be due to an increased percentage of granulocytes during the course of pregnancy (slope 1.37; p = 0.0003). In Wistar-rats, the WBC-count did not change during pregnancy, although percentage of granulocytes increased (slope 0.73; p = 0.0012) and percentage of lymphocytes decreased (slope −0.79; p = 0.0009) during the course of pregnancy. These changes in W-rats appeared to be less pronounced as compared to BBDP-rats, since we observed a trend towards significant different slopes (slope 1.37 vs. 0.73; p = 0.08 in granulocytes and slope −1.63 vs. −0.65; p = 0.06 in lymphocytes).

### Increased Th/Tc-ratio in pregnant BBDP-rats

**Figure 5:**
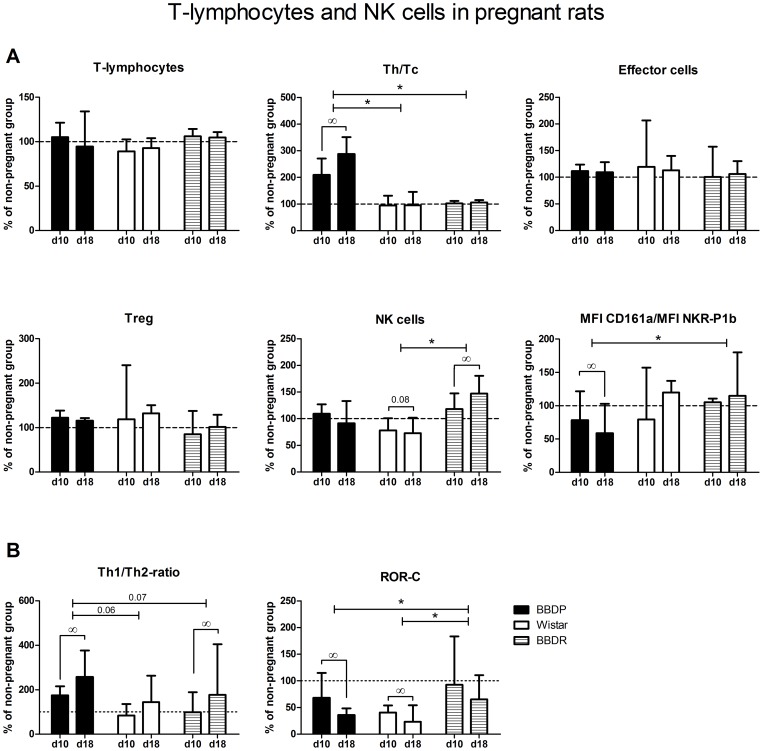
Lymphocyte subpopulations in pregnant rats. Values of day 10 or 18 of pregnancy are recalculated regarding the median values of non-pregnant animals (which was set at 100%) to express (as median (Q_1_–Q_3_)) pregnancy-induced changes. [A] The adaptations to pregnancy in percentage of T-lymphocytes, ratio of T-helper cells (Th) and cytotoxic T-cells (Tc), percentages of effector T-cells (Teff), regulatory T-cells (Treg), NK-cells and the ratio of MFI CD161a/NKR-P1b of NK-cells in all three strains during pregnancy. [B] The adaptations to pregnancy of the Th1/Th2-mRNA ratio and ROR-C mRNA expression in all three strains during pregnancy. d10/d18: day 10 or 18 of pregnancy. ∞slope significantly different from zero (Regression-analysis, ANCOVA, p<0.05). *slope significant different between the marked strains (Regression-analysis, ANCOVA, p<0.05).

As can be seen from figure 5A, little changes in T-lymphocyte subsets are observed in pregnant vs. non-pregnant animals in all three rat strains. Only, in BBDP-rats, Th/Tc-ratio increased during the course of pregnancy (slope 0.223; p = 0.019).

### Decreased stimulatory/inhibitory receptors on NK-cells in pregnant BBDP-rats

Although, in BBDP-rats the percentage of NK-cells was not affected by pregnancy, the MFI ratio CD161a/NKR-P1b decreased in BBDP-rats during the course of pregnancy (slope −0.783; p = 0.02). In W-rats, the percentage of NK-cells appeared to slightly decrease during the course of pregnancy (slope −0.044; p = 0.09), while the ratio MFI CD161a+/NKR-P1b+ remained stable.

### Pregnancy increased the Th1/Th2 ratio in BBDP-rats

The Th1/Th2-ratio increased during the course of pregnancy in BBDP (0.305; p = 0.045) and BBDR-rats (slope 0.061; p = 0.02), but not in W-rats, resulting in a trend towards a significantly steeper slope in BBDP vs. W-rats (p = 0.06; Fig. 5B). Expression of ROR-C similarly decreased during the course of pregnancy in both BBDP and W-rats.

### Increased ratio non-classical/classical monocytes during pregnancy

**Figure 6:**
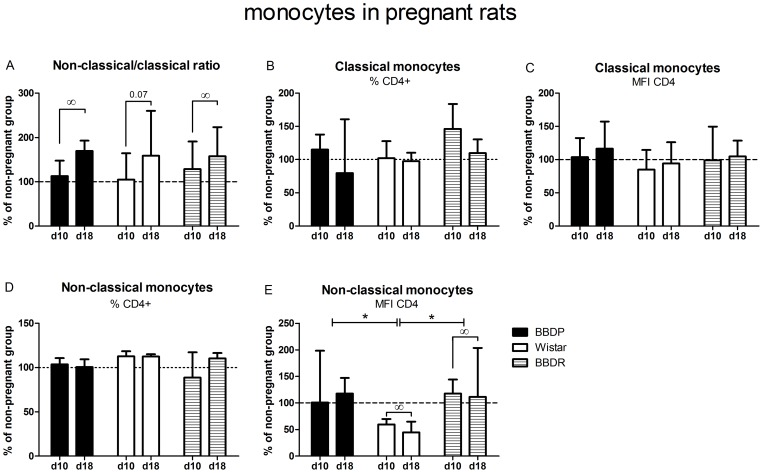
Monocyte subpopulations in pregnant rats. Values of day 10 or 18 of pregnancy are recalculated regarding the median values of non-pregnant animals (which was set at 100%) to express (as median (Q_1_–Q_3_)) pregnancy-induced changes. [A] The adaptations to pregnancy in the ratio non-classical/classical monocytes, [B] the percentage of CD4^+^ classical monocytes, [C] the MFI of CD4 on classical monocytes, [D] the percentage of CD4^+^ non-classical monocytes and [E] the MFI of CD4 on non-classical monocytes of each strain during pregnancy. d10/d18: day 10 or 18 of pregnancy. ∞slope significantly different from zero (Regression-analysis, ANCOVA, p<0.05). *slope significant different between the marked strains (Regression-analysis, ANCOVA, p<0.05).

As can be seen in figure 6, an increased ratio of non-classical/classical monocytes was found in all three strains during the course of pregnancy (BBDP: slope 0.043; p = 0.02, W: slope 0.022; p = 0.07, BBDR: slope 0.070; p = 0.002). Although the percentages of CD4^+^ classical or non-classical monocytes and CD4-expression on classical monocytes was not affected by pregnancy, CD4 expression on non-classical monocytes decreased during the course of pregnancy in W-rats (slope −0.51; p = 0.01), which was not observed in BBDP-rats. The slopes of BBDP and BBDR-rats were significantly different from the slope of W-rats (p = 0.003 and p = 0.002 respectively).

## Discussion

In the present study we used BBDP-rats to evaluate adaptations of the immune-response to pregnancy during type 1 diabetes under normoglycemic conditions. The BBDP-rat is a generally well accepted model for T1D and one of the most commonly used T1D models among rodents [25]. These animals show a mutation in the Gimap5 gene, which not only results in lymphopenia, but also in decreased numbers of regulatory T cells. This is in line with our findings (absolute numbers of lymphocytes and Treg are lower in BBDP-rats as compared with the other groups). These changes are important for the development of the autoimmunity and thus T1D [29]. We questioned whether due to the presence of the autoimmune disease in these BBDP-rats, the immunological adaptations to pregnancy would be different as compared to healthy rats, resulting in higher number of complications in the diabetic animals. Similar to T1D in human pregnancy, we found less favorable pregnancy outcome in BBDP-rats as compared to non-diabetic BBDR and W-rats. These complications coincided with a different immune-response in pregnant and non-pregnant BBDP-rats. The immune-response in non-pregnant BBDP-rats is characterized by decreased percentages of lymphocytes, increased percentages of Teff, Treg and NK-cells, an increased Th1/Th2-ratio and activated monocytes versus W and BBDR-rats. Furthermore, in BBDP-rats we found different pregnancy-induced adaptations as compared to W and BBDR-rats, i.e. an increased Th1/Th2-ratio, a decreased MFI CD161a/NKR-P1b ratio in comparison with the adaptation in W-rats. Moreover, in contrast to W-rats (in which monocytes were activated during pregnancy), we found no further activation of monocytes during pregnancy in BBDP-rats.

Despite the differences in immune-response between non-pregnant BBDP-rats and non-pregnant W and BBDR-rats (i.e. the immune-response at the start of pregnancy), all rat-strains are able to become pregnant. However, adverse fetal outcome in BBDP-rats was observed and characterized by lower fetal numbers, lower fetal weight of living fetuses, lower placental weight and increased numbers of resorptions versus our control rats. Interestingly, BBDR-rats also showed lower number of fetuses and lower fetal weight as compared to W-rats. This may coincide with the fact that also these rats showed different immunological adaptations to pregnancy as compared with W-rats, such as an increased Th1/Th2-ratio and lack of activation of non-classical monocytes.

Pregnancy outcome in women with T1D is also associated with adverse outcome [1], [2]. However, pregnancy outcome parameters of rats are not completely similar to human outcome parameters. For instance, we observed decreased fetal weight in T1D rat pregnancy, i.e. growth retardation, while human diabetic pregnancy is often accompanied by macrosomia. This difference between humans and rats is probably related to the fact that glucose levels in T1D rats were better controlled then in T1D human pregnancy. T1D in human pregnancy is associated with moderate hyperglycemic episodes, despite relatively good glycemic regulation [1], [5]. Indeed, previous studies using rat models for diabetes, showed increased pup weight, when glycemia was not well controlled [30], [31]. The increased numbers of resorptions in rat T1D pregnancy may be comparable to miscarriage in humans (rats do resorb fetuses, rather than abort them), which occurs more frequently in human T1D pregnancy. We found a slightly decreased number of implantations sites. A decreased number of implantation sites could indicate a diminished number of fertilized oocytes or a decreased number of fertilized oocytes that is able to implant. Immunological differences observed in our study between diabetic and non-diabetic animals could play an important role in development of the adverse pregnancy outcome in BBDP-rats [7], [32].

The increased Th1/Th2-ratio in non-pregnant BBDP-rats was further increased during pregnancy in contrast to W-rats, since these rats showed no changes in Th1/Th2-ratio during pregnancy. In human pregnancy, such an increased Th1/Th2-ratio is associated with recurrent miscarriage and fetal growth retardation [10], [15], [18]. Indeed, in diabetic rats, we found increased resorptions and decreased fetal weight. An upregulation of Th17 and decreased number/function of Treg are also associated with recurrent miscarriage and pre-eclampsia [33], [34]. In our study, the adaptations of Treg and ROR-C during pregnancy appeared to be the same for BBDP and W-rats. This may suggest that these cells do not play a role in fetal complications in BBDP-rats. However, it has been shown that the function of Treg in BBDP-rats is decreased [29], [35]. If this is also the case in pregnant BBDP-rats, such a decreased function could be involved in the fetal complications seen in our model.

The increased percentage of NK-cells in non-pregnant BBDP-rats, in contrast to W-rats, did not decrease during pregnancy. Although minor knowledge is available about peripheral NK-cells in rat pregnancy, healthy pregnant women showed reduced numbers of peripheral NK-cells [11], [36]. This is in line with the observations in W-rats. Moreover, increased numbers of peripheral NK-cells during human pregnancy have been associated with recurrent spontaneous abortion [37]. This may suggest that increased numbers of NK-cells in pregnant BBDP-rats may be involved in increased numbers of resorptions in these rats. The increased numbers of peripheral NK-cells in BBDP-rats may be due to impaired migration of these cells to the maternal uterus where they play an important role in preparing the uterine wall and vasculature for trophoblast-invasion [38]. This decreased migration of NK-cells in BBDP-rats could possibly lead to a less optimal trophoblast-invasion and result in smaller fetuses or more fetal resorptions. In rats, the receptors CD161a and NKR-P1b function as stimulatory and inhibitory receptors respectively [39]. Therefore, decreased CD161a/NKR-P1b expression ratio in BBDP-rats during pregnancy indicates a shift towards inhibitory receptors. Interestingly, it was suggested that an overexpression of inhibitory receptors was associated with impaired trophoblast-invasion and pre-eclampsia [14], [40]. However, further studies are needed to evaluate the role of these NK-cells in pregnancy of BBDP-rats.

In rats, monocyte subdivision into classical and non-classical monocytes is based on CD43 expression [41]. Classical monocytes (CD43^+^) are associated with extravasation to inflamed tissue where they develop into macrophages and help with pathogen clearance and wound healing [42]. Non-classical monocytes (CD43^++^) are thought to play a role in replenishment of resident tissue macrophages; they produce increased amounts of pro-inflammatory cytokines [42], [43]. Increased numbers of these monocytes are seen in inflammatory conditions [43]–[45]. Non-pregnant BBDP and BBDR-rats display a pro-inflammatory status as compared with W-rats, judging from their increased ratio of non-classical/classical monocytes. Also, the activational status of monocytes in these rats was increased, i.e. CD4 expression was decreased [28]. During pregnancy, however, the ratio non-classical/classical monocytes increased in all three rat strains as compared to non-pregnant rats, which is in line with a previous study from our lab [46]. Also the decreased expression of CD4 on non-classical monocytes in pregnant W-rats indicates activation of these cells. These observations corroborates the general accepted hypothesis that pregnancy is associated with generalized activation of the inflammatory response [47], [48]. However, since the ratio non-classical/classical monocytes in BBDP and BBDR-rats was already higher as compared to W-rats before onset of pregnancy, the similar increase of this ratio in all three rat-strains during pregnancy results in higher activation of monocytes during pregnancy in BB-rats as compared with W-rats. Such a further activation of the inflammatory response in human pregnancy is associated with pre-eclampsia, recurrent pregnancy loss and preterm labor [14]–[16].

In conclusion, this study has shown that the peripheral immune-response in non-pregnant BBDP-rats, i.e. at the start of pregnancy, is different compared with the control rats. We also observed different adaptations of the immune-response to pregnancy in these BBDP-rats. Since aberrant immunological adaptations to pregnancy are associated with maternal and perinatal complications, it may be suggested that the observed decreased number of fetuses, lower fetal weight, lower placental weight and more resorptions in BBDP-rats are a consequence of these different immunological adaptations. This suggestion corresponds with data on human pregnancy in patients with other autoimmune diseases, such as rheumatoid arthritis or systemic lupus erytematosus, in which the presence of the autoimmune disease results in complications, such as recurrent miscarriages, decreased birth weight and preterm birth [49]–[51]. Therefore, it may be postulated that the immune process in patients with T1D is, besides hyperglycemia, also responsible for the adverse pregnancy outcome. The present study was an observational study in rats, performed to substantiate our hypothesis that factors associated with the presence of an autoimmune disease are involved in pregnancy complications observed in diabetic pregnancies. Since our data suggest different immune responses between control and T1D rats, further mechanistically insights into the role of various immune cells in implantation, placental and fetal development in healthy and diabetic pregnancy is necessary, as well as conformation of these results in human pregnancy.
